# Biogenic zinc oxide nanoregulator determines the quantitative analysis of morpho‐anatomical and antioxidant capacity in *Lactuca sativa L*.

**DOI:** 10.1002/fsn3.4261

**Published:** 2024-08-18

**Authors:** Murtaza Hasan, Tuba Tariq, Ghazala Mustafa, Emad A. A. Ismail, Fuad A. Awwad, Mehrnaz Hatami

**Affiliations:** ^1^ Department of Biotechnology, Faculty of Chemical and Biological Sciences The Islamia University of Bahawalpur Bahawalpur Pakistan; ^2^ Department of Plant Sciences, Faculty of Biological Sciences Quaid‐i‐Azam University Islamabad Pakistan; ^3^ Department of Quantitative Analysis, College of Business Administration King Saud University Riyadh Saudi Arabia; ^4^ Department of Medicinal Plants, Faculty of Agriculture and Natural Resources Arak University Arak Iran; ^5^ Institute of Nanoscience and Nanotechnology Arak University Arak Iran

**Keywords:** anatomy, bio‐fortification, nano‐biocatalys, nanofertilizer, xylem and phloem

## Abstract

Zinc oxide nanoparticles (ZnO NPs) can possibly serve as a pervasive source of essential nutrient zinc in agricultural crops in the future. The major environmental concerns with ZnO NPs might be their toxicity and incorrect dosage, which might lead to crop damage and environmental pollution. Interplay between ZnO NPs and *Lactuca sativa* (*L. sativa*) will be of interest, yet little is known about apropos interaction of these two, which will assist in optimizing the dose of ZnO NPs for their commercial use in agriculture. The current study aimed to investigate the growth, anatomical, and antioxidative responses of *L. sativa* against ZnO NPs and zinc acetate. ZnO NPs were foliar sprayed with concentrations of 0, 25, 50, and 100 ppm. ZnO NPs remarkably promoted *L. sativa* growth, leaf water content, and biomass accumulation; however, they declined root growth. The foliar spray of ZnO NPs improved the thickness of the stem hypodermis, cortex, pericycle, and phloem, while decreasing the stem diameter, thickness of the epidermis, and number of vascular bundles. ZnO NPs rigorously declined the cell area of xylem but slightly improved it in phloem. Unlike stem cells’ anatomical responses to ZnO NPs, the root cells behaved otherwise. Overall, the antioxidative activity of *L. sativa* considerably improved at 25 ppm concentration and decreased at 100 ppm. Generally, low and medium concentrations of ZnO NPs promoted plant morphological, anatomical, and antioxidant traits, while higher doses inhibited the same traits. Contrary to this, Zn acetate displayed severe toxic effects on almost all studied anatomical traits.

## Graphical abstract

ZnO NPs improved the thickness of the stem hypodermis, cortex, pericycle, and phloem. Biochemical and anatomical changes showed improvements in lettuce growth. Anti‐oxidative response of lettuce against ZnO NPs and zinc acetate was assayed.

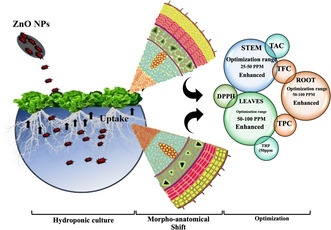

## 1 INTRODUCTION

The world's human population is expected to cross 9.8 billion individuals by the end of 2050 and 11.2 billion at the end of this century (WPP, United Nations, Department of Economic and Social Affairs, Population Division, 2019). It is widely known that world agricultural productivity should increase to feed the fast‐growing population (Prabhakar, 2021; Qiu et al., 2019). The population of developing countries faces daily food shortages as a result of climate change, whereas within the developed world, there is always a food surplus (Singh & Sengar, 2020). The agricultural food production of a country has great significance because it has been one of the first drivers of the economy (Osinowo et al., 2021). Climate change and resource constraints are quickly growing, and the world population is putting unexampled pressure on food demand, which forces us to look for innovative techniques for efficient resource utilization and to enhance crop productivity (Fukase & Martin, 2020; Rocha et al., 2023). There is an increasing demand by consumers for nutrient rich foods that improve physical performance and scale back malnutrition and disease risks (Anzani et al., 2020).

Lettuce (*L. sativa* L.), commonly found in every corner of the world, has an annual global consumption of about 26 million tons (Armas et al., 2017). This vegetable is a source of fiber, minerals, and vitamins A, B1, B2, B6, and C, in addition to possessing laxative, drug, and lenitive properties. There are many ways to improve the quantity and quality of *L. sativa*, like agronomic management (Shrestha & Lindsey, 2019), breeding techniques (Krieger et al., 2020), tissue culture, biotechnology (Hunt et al., 2009), and nanotechnology (Hasan, Ullah, et al., 2018; Saif et al., 2021; Zulfiqar et al., 2019). Careless and risky chemical usage for crop nutrition has increased soil toxicity and water pollution and lowered crop productivity, soil biological activity, soil assortment, and bird's’ diversity and population (Tudi et al., 2021). The fertilizers are important for plant growth and development; most of the applied fertilizers leached into the soil and were not uptake by plants due to degradation by reactions, fixation in the soil (Hasan et al., 2014), quality and formulation of the used fertilizer, soil composition and pH (Mustafa & Komatsu, 2022; Shehzad & Mustafa, 2023; Zaheer et al., 2023), decomposition rate of the fertilizer, suppression by other nutrients, and toxicity (Chen et al., 2022). Additionally, repeated application of typical fertilizers at a higher rate and for a protracted amount within agriculture fields has caused major environmental problems worldwide (Bindraban et al., 2020). Significant use of elemental fertilizers has become the most important phylogeny factor leading to world‐wide eutrophication issues in fresh bodies and coastal ecosystems (Romanelli et al., 2020). Thus, imperative analysis must be developed to reduce nutrient losses in fertilization and extend crop yield through the exploitation of the latest technologies (Ragaei & Hassan Sabry, 2014). To combat this drawback, the development of more practical, non‐persistent, and slow‐releasing fertilizers like controlled unleash formulations is required. Thus, the optimum metallic contents of all essential nutrients, including zinc, are desirable for the healthier growth of plants in a controlled way (Hasan et al., 2021; Karimzadeh Asl et al., 2018).

In agriculture, nanoscience will contribute to improving crop yield through the introduction of highly efficient alternative agrochemicals (Hasan, Zafar, et al., 2020; Huang et al., 2023; Mustafa et al., 2022; Saif et al., 2023), fertilizers, hormones, or growth agents (Khalid et al., 2022). Nanoparticles with encapsulated fertilizers are used for the slow and sustained unleashing of nutrients (Elsayed et al., 2022; Verma et al., 2022). Nano‐fertilizers can cut back on soil and water exploitation with chemical fertilizers and are more practical and environment‐friendly (Mustafa et al., 2020). These nanostructures also have higher reactivity (Huang et al., 2022), bioavailability (Dang et al., 2014), bioactivity, and surface effects (Zafar et al., 2021). Many researchers are trying different varieties of NPs to be helpful in many alternative applications (Luo et al., 2020; Wu et al., 2021). Among the prevailing metal NPs, ZnO NPs have gained the awareness of researchers due to their distinctive effective properties like antimicrobial (Hasan, Altaf, et al., 2020), antifungal, antiviral, anticancer (Cao et al., 2020), anti‐diabetic, and anti‐biofilms (Hasan, Zafar, et al., 2020). In this study, the *L. sativa* plant is chosen for its quick rate of growth. The present study aims to examine the consequences of ZnO NPs and metallic element zinc acetate on *L. sativa* growth and biomass accumulation in hydroponic culture, investigate the morphological and anatomical changes of *L. sativa* at different concentrations of ZnO NPs, explore the antioxidative response of *L. sativa*, and compare whether ZnO NPs or metallic element acetate is better for their use as a Zn source in *L. sativa*.

## 2 MATERIALS AND METHODS

### 2.1 Preparation of *Withania coagulans* extract and green synthesis of ZnONPs

*Withania coagulans* fruits were collected from the wild desert area of the Islamia University of Bahawalpur, which is located at the periphery of the Cholistan Desert. Fruits of *W. coagulans* were washed with tap water once and then rewashed with distilled water twice to remove contaminants before sun drying for the next three weeks. After drying, the fruits were crushed into small, granulated pieces by using a pestle and mortar. At least 10 g of granulated fruit of *W. coagulans* were poured into 100 mL of distilled water in a 500 mL beaker, covered with an aluminum foil, and heated on a hotplate for 2 h at 72°C. The mixture was filtered using Whitman filter paper No. 1, and the filtered extract was poured into a conical flask and stored at 4°C. The biological synthesis of zinc NPs was carried out in the laboratory by a previously elaborated method with little modification (Akbar et al., 2018; Hasan et al., 2013).

### 2.2 Growth of lettuce in hydroponic culture

The seeds of *L. sativa* were taken from Sky Seed Company in Lahore, Punjab, Pakistan. These seeds were grown in a seedling tray with coconut peat as a solid organic medium (Figure S1). This organic medium was sprinkled with enough water (Figures S2–S5). Then, this seedling tray was kept in a hydroponic tray to ensure moisture and water accessibility to seeds for maximum germination, and then it was kept in a sunny area with a temperature between 8.3 and 26.6°C. Ten identical seedlings were transferred to pots at the age of 12 days. Each pot was kept in a separate plastic reservoir carefully, which acts as a reservoir of nutrients. These plastic reservoirs were filled with 1000 mL of Hoagland's solution. To ensure that the plant root and solid hydroponic medium are in contact with the liquid, the liquid medium was adjusted to 1000 mL solutions of zinc oxide NPs having different concentrations of 0, 25, 50 and 100 ppm in distilled water were prepared by using ultrasonic vibrations at 30 kHz for 30 min. Daily, every test plantlet was supplied with 15 mL of zinc oxide NPs (ZnO NPs) solution of each concentration through foliar spray. This supply of ZnO NPs was carried out constantly for 35 days, along with control and zinc acetate concentrations of 0.01 M. All plants were kept and maintained on a 14 h light/10 h dark cycle, and the temperature was about ~22°C.

### 2.3 Plant morphological observations

At the end, *L. sativa* plants were harvested and washed using MilliQ water. *L. sativa* growth and morphological changes were recorded for plant height (cm), shoot length (cm), root length (cm), leaf area (cm^2^), number of leaves per plant, fresh weight of plant (g), dry weight of plant (g), and leaf water content (Kučerová et al., 2021). All the samples were, without delay, frozen for further analysis at −80°C.

### 2.4 Anatomical observations of plant parts

The section cutting of *L. sativa* plants was done, and the sections were treated with a single staining dehydration process (safranine) to prepare permanent slides and then observed under a microscope (Barbosa et al., 2010). A serial dehydration solution was used for staining. Solutions were prepared with alcohol at 30%, 50%, 70%, and 90% (absolute alcohol). Xylene was used for the cleaning of cross sections of *L. sativa*. Canada balsam was used for the mounting purpose. Fine cross sections of the root, stem, and leaves of *L. sativa* plants were taken and preserved by using the single staining technique. Photographs were taken in the laboratory from a microscope by using a 24 mega pixel camera in the plant analysis laboratory at the Institute of Biochemistry and Biotechnology, the Islamia University of Bahawalpur, Punjab, Pakistan. Stem anatomical characterization included diameter of stem, thickness of epidermis (μm), thickness of hypodermis (μm), thickness of cortex (μm), thickness of pericycle (μm), thickness of xylem (μm), thickness of phloem (μm), thickness of pith (μm), and number of vascular bundles. Cell areas of stem tissues were measured for the epidermal cell area (μm), hypodermal cell (μm), cortex cell area (μm), pericycle cell area (μm), xylem cell area (μm), phloem cell area (μm), pith cell area (μm), sclerenchyma cell area (μm), parenchyma cell area (μm), and collenchyma cell area (μm). Root anatomical characterization included root diameter, thickness of epidermis (μm), thickness of cortex (μm), thickness of pericycle (μm), thickness of hypodermis (μm), thickness of xylem (μm), thickness of phloem (μm), thickness of pith (μm), number of modularly rays, and number of vascular bundles. Cell area of root tissues were measured for the Epidermis cell area (μm), cortex cell area (μm), endodermis cell area (μm), xylem cell area (μm), phloem cell area (μm), sclerenchyma cell area (μm), parenchyma cell area (μm), and collenchyma cell area. Leaves anatomical characterization included thickness of cuticle (μm), thickness of mid rib (μm), thickness of lamina (μm), thickness of abaxial epidermis (μm), thickness of adaxial epidermis (μm), thickness of palisade (μm), thickness of spongy (μm), thickness of xylem (μm), thickness of phloem and number of vascular bundles. The cell area of leaf tissues was measured for the leaf area (cm), abaxial cell area (μm), adaxial cell area (μm), palisade cell area (μm), spongy cell area (μm), xylem cell area (μm), phloem cell area (μm), sclerenchyma cell area (μm), parenchyma cell area (μm), and collenchyma cell area (μm).

### 2.5 Determination of antioxidative potential

The leaves of *L. sativa* were shade dried and then ground to a fine powder by using a mortar and pestle. The suspension was made with dimethyl sulfoxide (DMSO) in an Eppendorf tube at 20 mg/mL. The Eppendorf tubes were stored for 48 h at room temperature and then centrifuged for 3 min at 5000 rpm. The supernatant was used to determine the enzymatic antioxidant activities (DPPH‐based free radical scavenging activity, total antioxidant potential, and total reducing power) and non‐enzymatic antioxidant activities (phenolics and flavonoids). To determine the free radical scavenging activity, a 2,2‐diphenyl‐1‐picryl hydrazyl (DPPH) reagent was used (Yildiz et al., 2012). In brief, 190 μL of DPPH (0.004% w/v in methanol) was mixed with a plant extract of 19 μL. The mixture was incubated for 1 h in a dark place. The optical density at 515 nm was measured using a microplate reader. As a positive standard, ascorbic acid was used, while as a negative control, DMSO was used. The following formula was used to calculate the percent inhibition. Percent inhibition of samples = % free radical scavenging potential = (1 – Abs/Abc) × 100 Where Abs and Abc indicate the DPPH solution's absorption with the sample and the negative control's absorbance, respectively. Total antioxidant activity was measured by a modified method (Jiráková et al., 2020). The absorbance was measured by microplate reader at 695 nm. Instead of test samples, DMSO (100 μL) was used as a control and for the standard curve, ascorbic acid was used as a positive control was used. The ensuing total antioxidant capacity (TAC) was articulated as μg ascorbic acid equivalent per mg FW (μg AAE/mg FW). According to the formerly described method, the reduction potential of the samples was determined. Ascorbic acid was taken as a positive control, and their results were articulated as μg of ascorbic acid in equivalent/mg of FW (μg AAE/mg FW). The phenolic content was measured according to standard protocol. Blank (DMSO) and the standard used (gallic acid in DMSO) were both simultaneously run as controls. A curve was made in parallel. The TPC was measured as μg of gallic acid equivalent/mg FW (μg GAE/mg FW). The total flavonoid content was determined by the formerly described method by Ul‐Haq et al. (2012). The absorbance was taken at 415 nm by a microplate reader. The curve was made by quercetin as a standard at 0–40 μg/mL, and the flavonoid content was measured in μg quercetin equivalent/mg of FW (μg QE/mg FW).

### 2.6 Statistical analysis

There were three independent experiments carried out for all experiments, and the results were illustrated in mean value ± standard deviation (SD).

## 3 RESULTS AND DISCUSSION

### 3.1 *Lactuca sativa* growth, biomass accumulation, and water content

**FIGURE 1:**
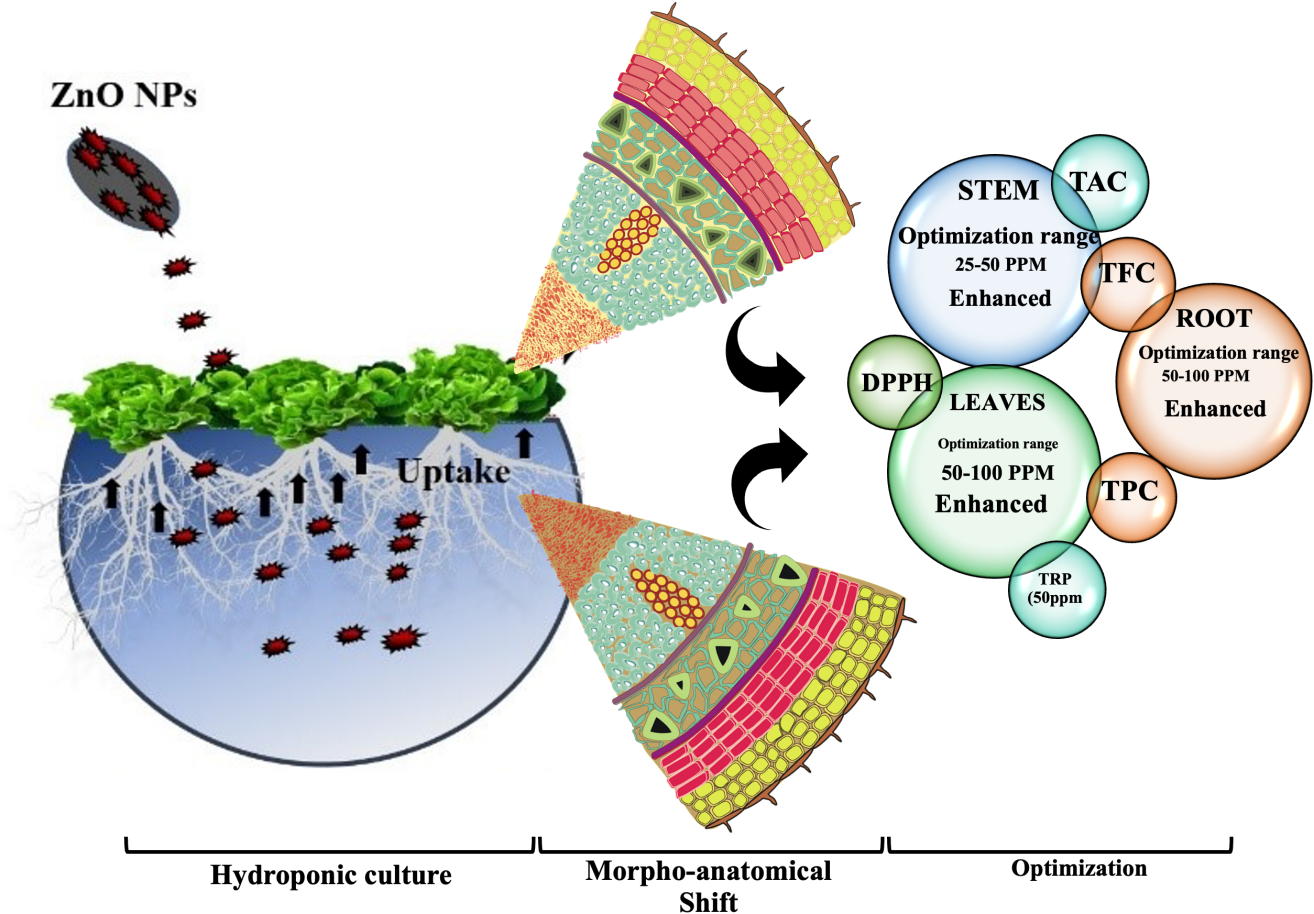
Application of ZnO NPs in hydroponic culture, regulating morpho‐anatomical shifts, and their optimization in *Lactuca sativa* L.

The overall application of ZnO NPs in hydroponic culture, regulating morpho‐anatomical shifts, and their optimization level in *L. sativa* plant are presented in Figure 1. The application of ZnO NPs noticeably improved *L. sativa* plant height, leaf area, and number of leaves per plant as compared to control and Zn acetate (Figure S6a–c). The effect of ZnO NPs was positive and significant for shoot length, while it was negative and non‐significant for root length for all concentrations except for 50 ppm, which showed a slighter increase in root length as compared to control (Figure S7). So, shoot length and root length presented an inverse relationship against the foliar application of ZnO NPs. The application of ZnO NPs displayed a remarkable improvement in leaf water content and fresh and dry biomass accumulation (Figure S8a,c). In crux, the foliar spray of ZnO NPs had a considerable growth‐promoting effect on all studied plant morphological traits except for root length, which confirmed both inhibitory and promotive responses based on the concentration of the NPs. Thus, we may conclude from the ANOVA results that the effectiveness of the ZnO NPs significantly varied based on the concentration of the NPs and the studied morphological trait. An increase in the concentration of ZnO NPs gradually improved significantly leaf area (240%), leaf water content (425%), and fresh and dry biomass accumulation (535–418%) and has shown the maximum values for these traits at 100 ppm compared with control. Among different concentrations of ZnO NPs, the 50 ppm brought the maximum improvement in plant height (37%) and root length (10%), while it showed the least improvement in the number of leaves per plant. Astonishingly, the application of all concentrations of ZnO NPs has significantly improved the shoot length by 70% as compared to the control. Current work showed better results as compared to previous reports regarding the use of nanoparticles for promoting the morphological characteristics of crops and their end products (Hasan et al., 2023; Hasan et al., 2024; Javed et al., 2024; Mahmood et al., 2023; Zafar et al., 2024).

### 3.2 Anatomical responses of *L. sativa* stem to applied ZnO NPs

**FIGURE 2:**
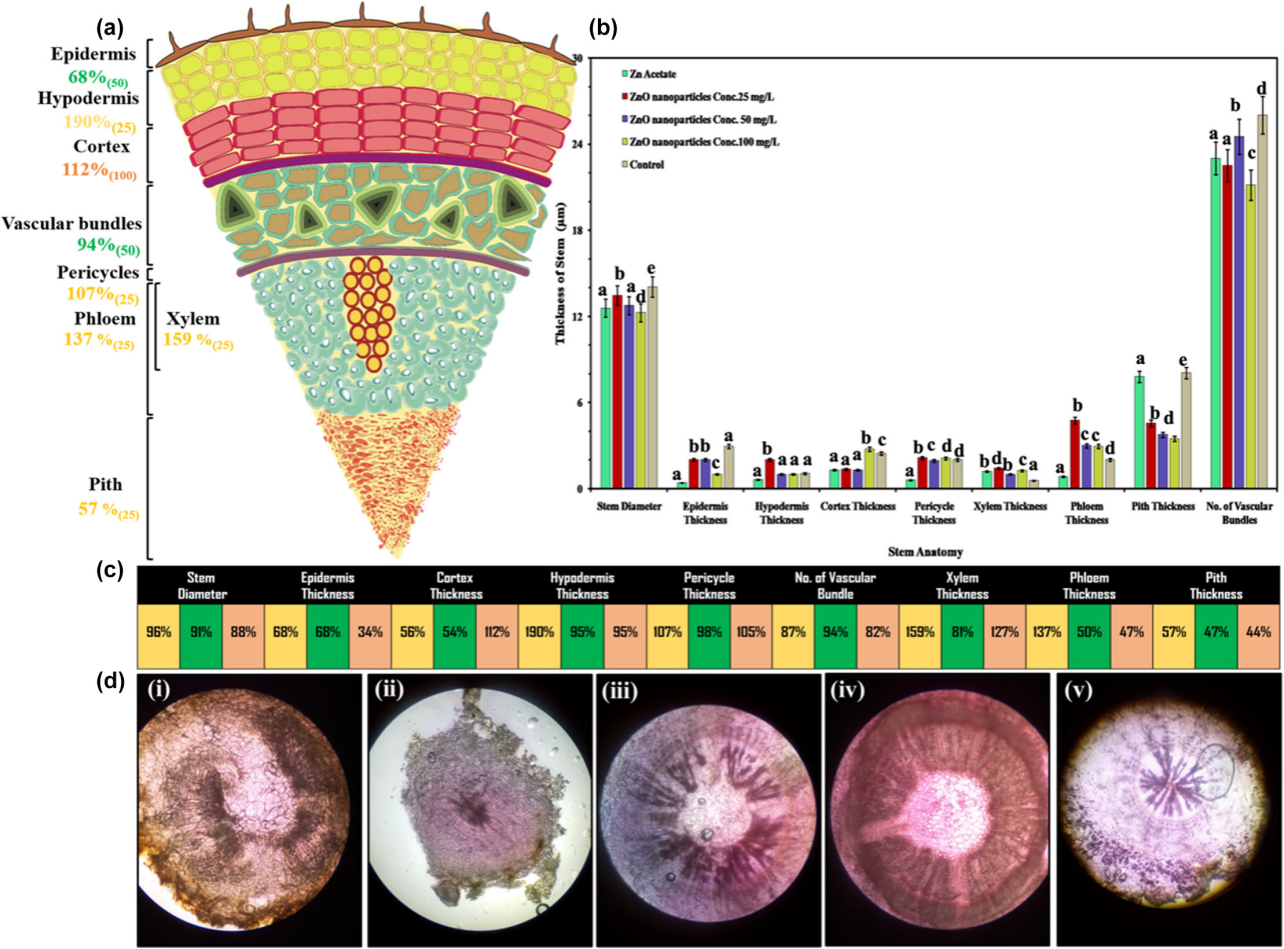
Effect of ZnO NPs on thickness of *Lactuca sativa* L stem against different concentrations: (a) represent a 50 ppm optimized value; (b) graphic shifts among controls; (c) elaboration of numerical significance changes; (d) cross‐sectional anatomical changes in different treatments at 1500×. The mean values at each point with different letters are significantly different according to ANOVA (*n* = 5, *p <* .05).

Starting with anatomical analysis of vegetative parts from stems, stem anatomical studies included the thickness and area of numerous stem cells. The application of ZnO NPs reported both enhancing and inhibitory effects on stem anatomical traits (Figure 2a–d). The ZnO NPs improved stem cell hypodermis thickness, cortex thickness, pericycle thickness, and phloem thickness, while they declined stem diameter, epidermis thickness, pith thickness, and the number of vascular bundles (Figure 2a). The ANOVA results reveal interactions between control, positive control, and all ZnO NPs treatments. The application of 25 ppm ZnO NPs has significantly promoted hypodermis thickness by 81%, pericycle thickness by 11%, xylem thickness by 160%, and phloem thickness by 110%, while 100 ppm improved cortex thickness by 18% as compared to control (Figure 2b).

**FIGURE 3:**
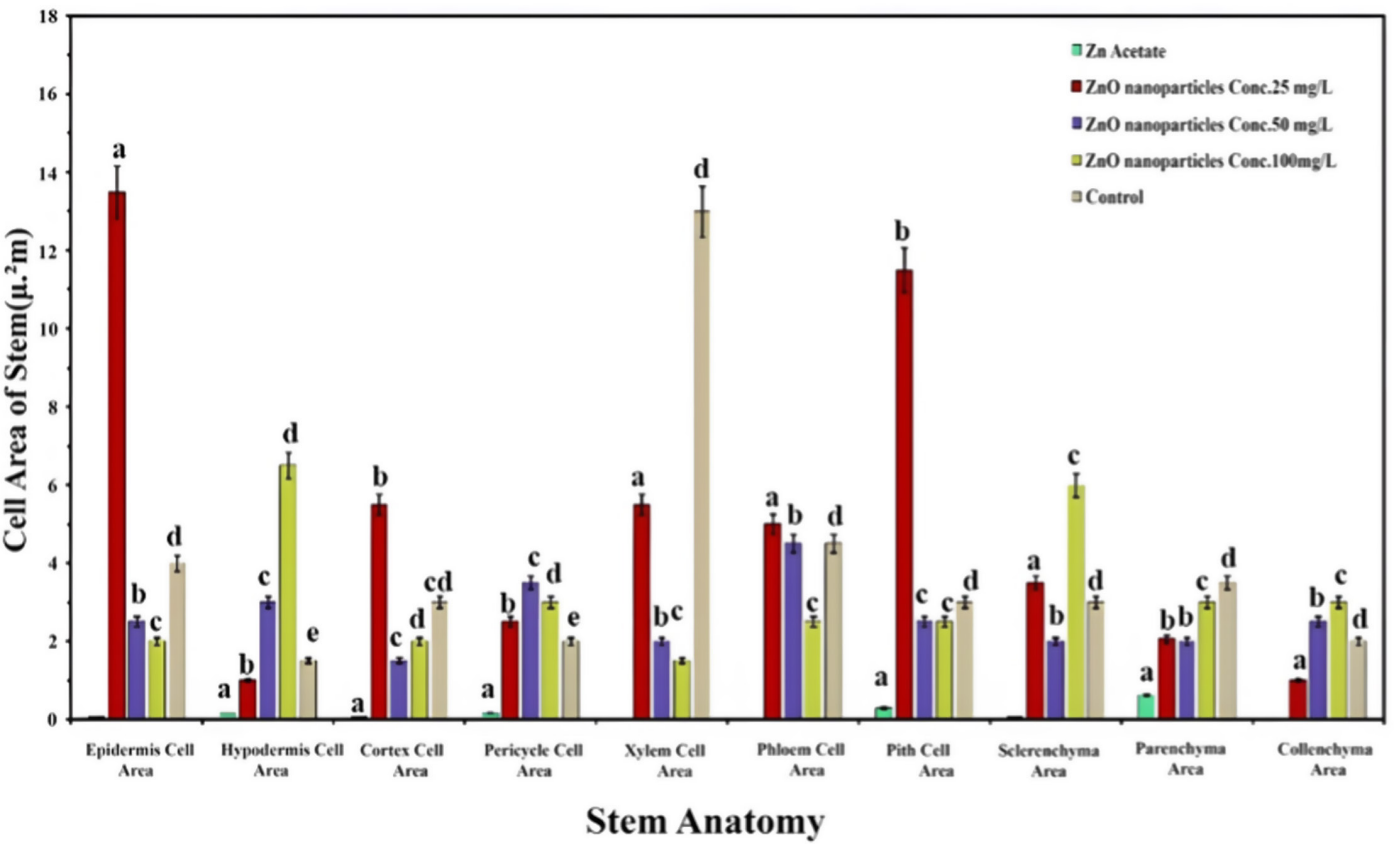
Effect of ZnO NPs on *Lactuca sativa* L cells in different areas of stem cells. The mean values at each point with different letters are significantly different according to ANOVA (*n* = 5, *p <* .05).

On the contrary, all concentrations of ZnO NPs proved toxic and declined significantly in stem diameter by −7%, pith thickness by −43%, and number of vascular bundles by only −3%. The toxicity levels worsen with an increase in the concentration of ZnO NPs from 25 ppm to 100 ppm for these traits. Microscopic studies displayed a significant promotive effect of ZnO NPs on all examined stem cell areas except for the xylem and parenchyma cell areas, which have shown a negative response (Figure 2d). However, the tabulation part of Figure 2c shows the different concentrations of ZnO NPs, such as 25 ppm represented with yellow box, 50 ppm with green box, and 100 ppm with light pink box. Furthermore, we have also observed that the application of ZnO NPs rigorously declined xylem cell area (−61%) and marginally decreased parenchyma cell area (14%) as compared to control. Also, the ANOVA results reveal interactions among different concentrations of ZnO NPs: 25 ppm extensively and significantly promoted epidermis cell area (237%), cortex cell area (63%), phloem cell area (06%), and pith cell area (258%); 50 ppm promoted pericycle cell area (65%); and 100 ppm stimulated hypodermis cell area (370%), sclerenchyma cell area (93%), and collenchyma cell area (42%), as shown in Figure 3.

### 3.3 Anatomical responses of the *L. sativa* root to applied ZnONPs

**FIGURE 4:**
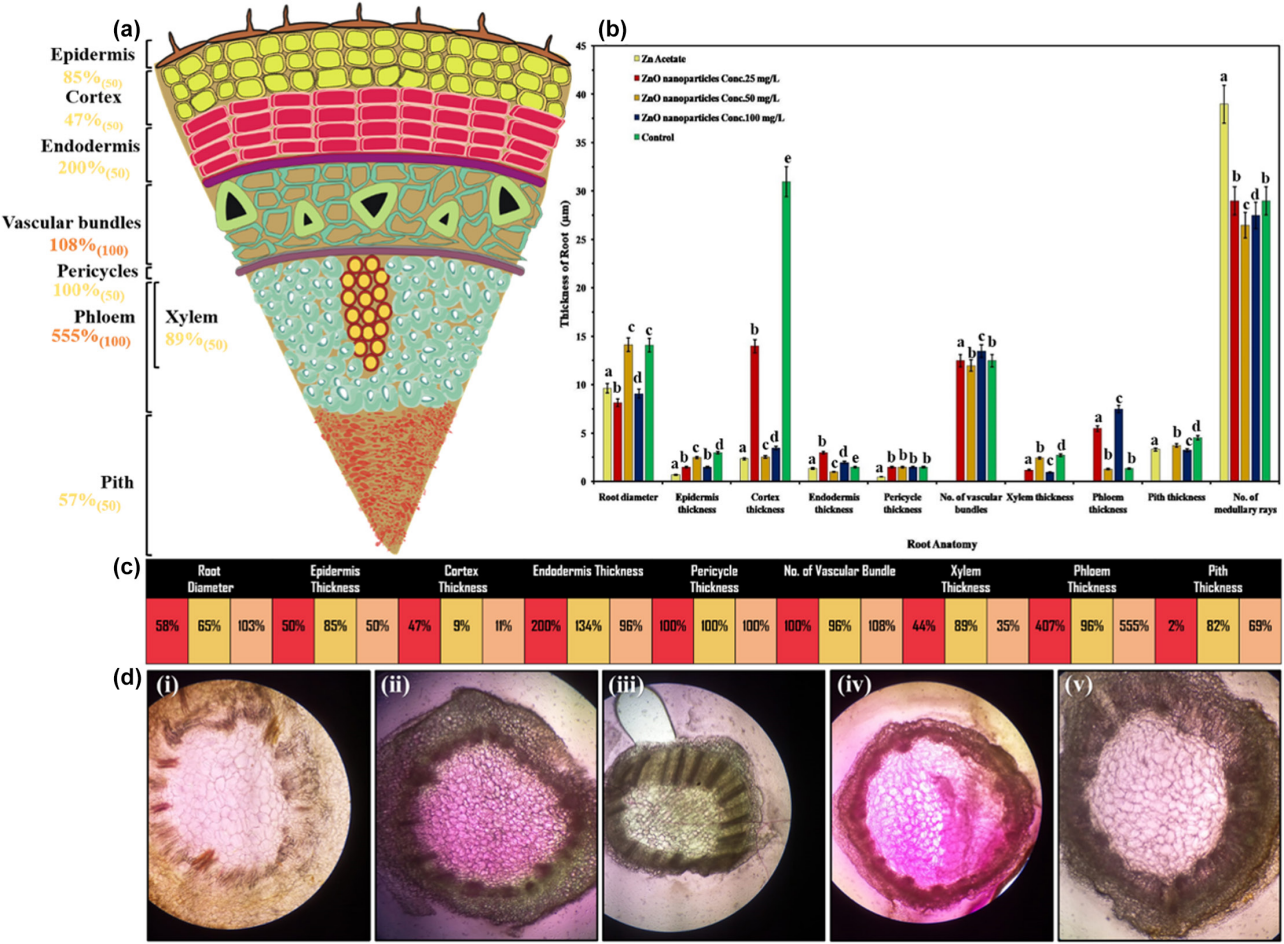
Effect of ZnONPs on thickness of *Lactuca sativa* L root against different concentrations: (a) represent a 50 ppm optimized value; (b) graphic shifts among controls; (c) elaboration of numerical significance changes; (d) cross‐sectional anatomical changes in different treatments at 1500×. The mean values at each point with different letters are significantly different according to ANOVA (*n* = 5, *p <* .05).

In the next section, Thickness of root cells (μm) studies included root diameter, epidermis, cortex, endodermis, pericycle, xylem, phloem, pith, number of vascular bundles, and number of medullary rays (Figure 4a). Unlike stem cells, there are anatomical responses to application of ZnO NPs in the root cells. The 50 ppm concentration of ZnO NPs produced the maximum diameter of root that was almost equal to the control treatment. Similarly, the thickness of the pericycle remained unchanged with or without the application of ZnO NPs. The thickness of epidermis, cortex, xylem, and pith cells significantly declined with all levels of ZnO NPs. However, an ANOVA test was applied to explore the effect of different treatment interactions and their comparative studies with control plants. The minimum concentration of ZnO NPs (25 ppm) has significantly improved the thickness of the endodermis by 40%, while the maximum concentration (100 ppm) has significantly stimulated the number of vascular bundles by 8% and phloem thickness by 300%. Unexpectedly, the application of Zn acetate improved the number of medullary rays by 35%, as shown in Figure 4b. However, the tabulation part of Figure 4c shows the different concentrations of ZnO NPs, such as 25 ppm represented with red box, 50 ppm with yellow box, and 100 ppm with light pink box. Furthermore, the microscopic study also reveals remarkable changes in *L. sativa* L due to variations in the concentration of NPs. The cell areas and cell structure clearly show difference, which strongly influence the physiological parts of the plant (Figure 4d).

**FIGURE 5:**
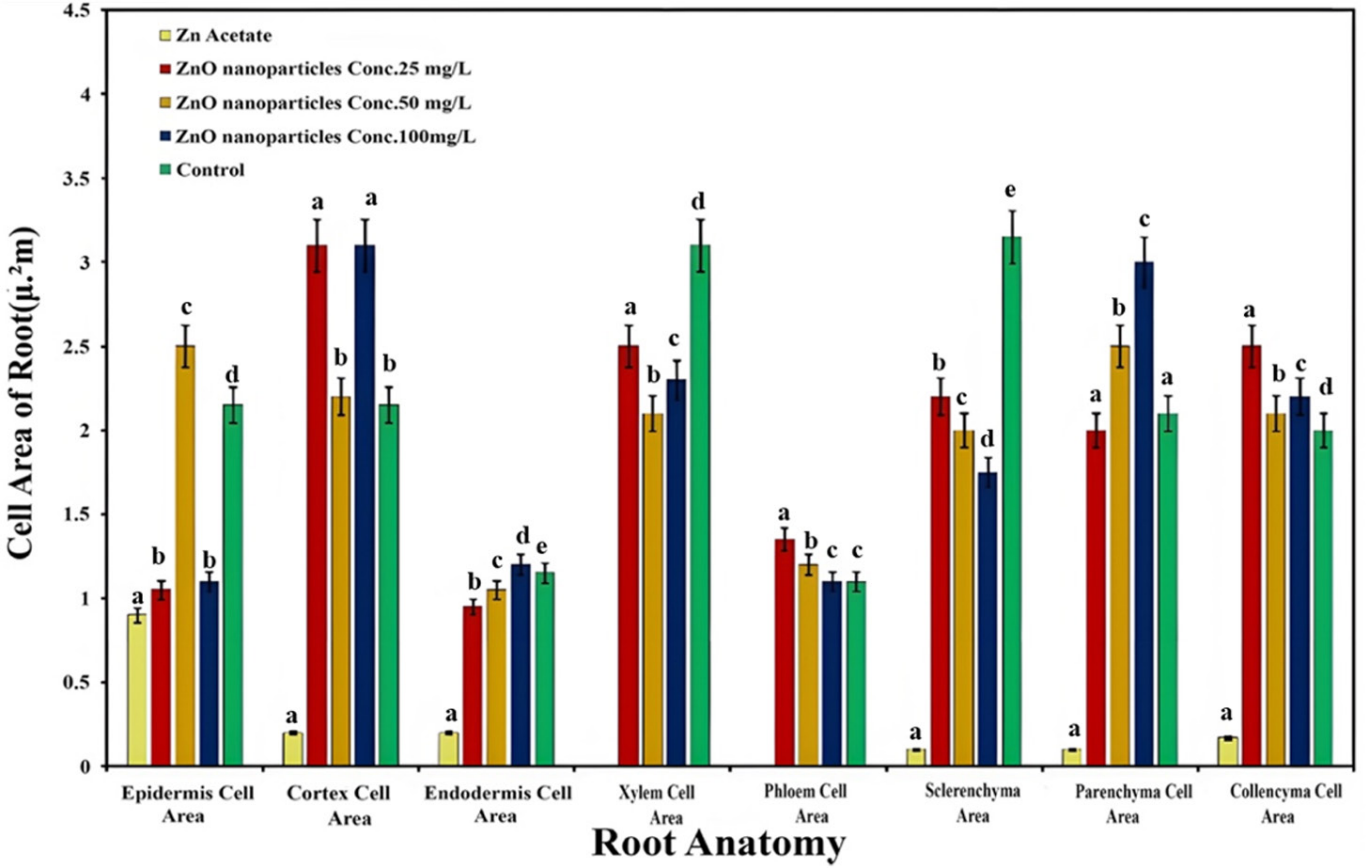
Effect of ZnONPs on the cell area of *Lactuca sativa* L cells in different areas of root cells. The mean values at each point with different letters are significantly different according to ANOVA (*n* = 5, *p <* .05).

Root cell area (μm^2^) studies reported a mixed trend in the applied concentration of ZnO NPs (Figure 5). The application of ZnO NPs gradually increased the cell area of the endodermis and parenchyma, along with the increase in the concentration of NPs, while the response of other traits was irregular. Among different concentrations of ZnO NPs, an ANOVA test was used to find that 25 ppm of ZnO NPs significantly expanded cell area of the phloem by 20% and the collenchyma by 26%, while 50 ppm of ZnO NPs extended the cell area of epidermis by 19%. The maximum concentration of ZnO NPs (100 ppm) significantly augmented the cell area of the endodermis by 5% and the parenchyma by 40%. Both low and high concentrations of ZnO NPs produced approximately the same cortex cell area, but it was significantly higher than the control. However, an ANOVA test has shown that the negative control group application of Zn acetate strongly displayed severe toxic effects on all root cell area traits, which clearly indicates the oxidative response.

### 3.4 Anatomical responses of *L. sativa* leaf to applied ZnO NPs

**FIGURE 6:**
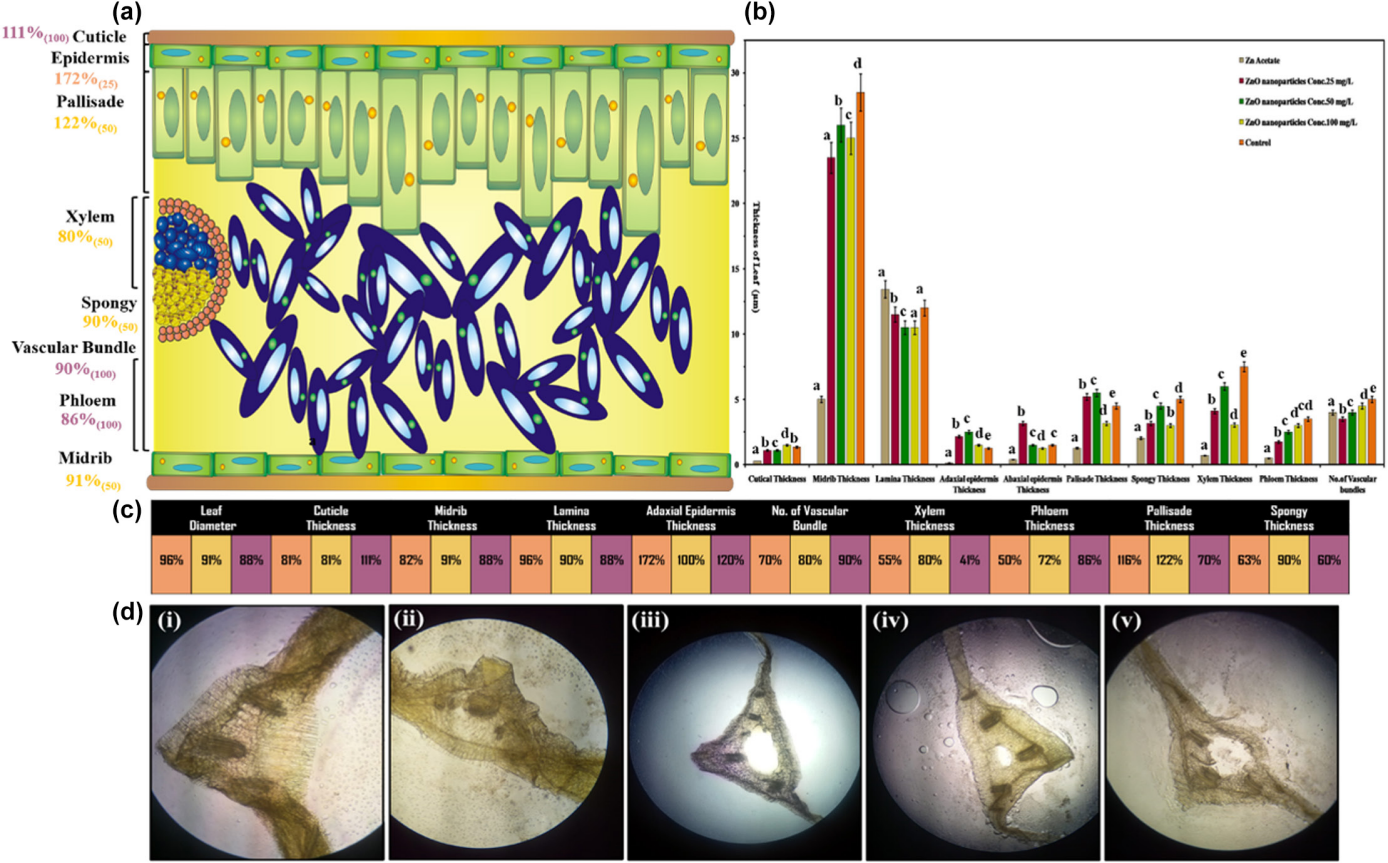
Effect of ZnO NPs on thickness of *Lactuca sativa* L leaf against different concentrations: (a) represent a 50 ppm optimized value; (b) graphic shifts among controls; (c) elaboration of numerical significance changes; (d) cross‐sectional anatomical changes in different treatments at 1500×. The mean values at each point with different letters are significantly different according to ANOVA (*n* = 5, *p <* .05).

Figure 6 illustrates the response of *L. sativa* leaf thickness parameters to applied ZnO NPs in hydroponic culture. The use of ZnONPs slightly improved cuticle thickness at 100 ppm concentration, while the application of Zn acetate reported the maximum lamina thickness (Figure 6a).

From the ANOVA test, it was found that the lower concentration of ZnO NPs significantly increased the thickness of the abaxial epidermis by 51%, while the medium concentration favored the thickness of the adaxial epidermis and palisade by 58% to 20% simultaneously. On the contrary, the foliar application of ZnO NPs discouraged the thickness of the midrib, spongy cells, xylem, phloem, and number of vascular bundles as compared to the control (Figure 6b). However, the tabulation part of Figure 6c shows the different concentrations of ZnO NPs, such as 25 ppm represented with orange box, 50 ppm with yellow box, and 100 ppm with purple box. Furthermore, microscopic and histological studies of the leaf were also carried out to investigate the real situation happening within the cells by the application of ZnO NPs with different concentrations and to know about the prohibitory and stimulating influence on *L. sativa* (Figure 6d).

**FIGURE 7:**
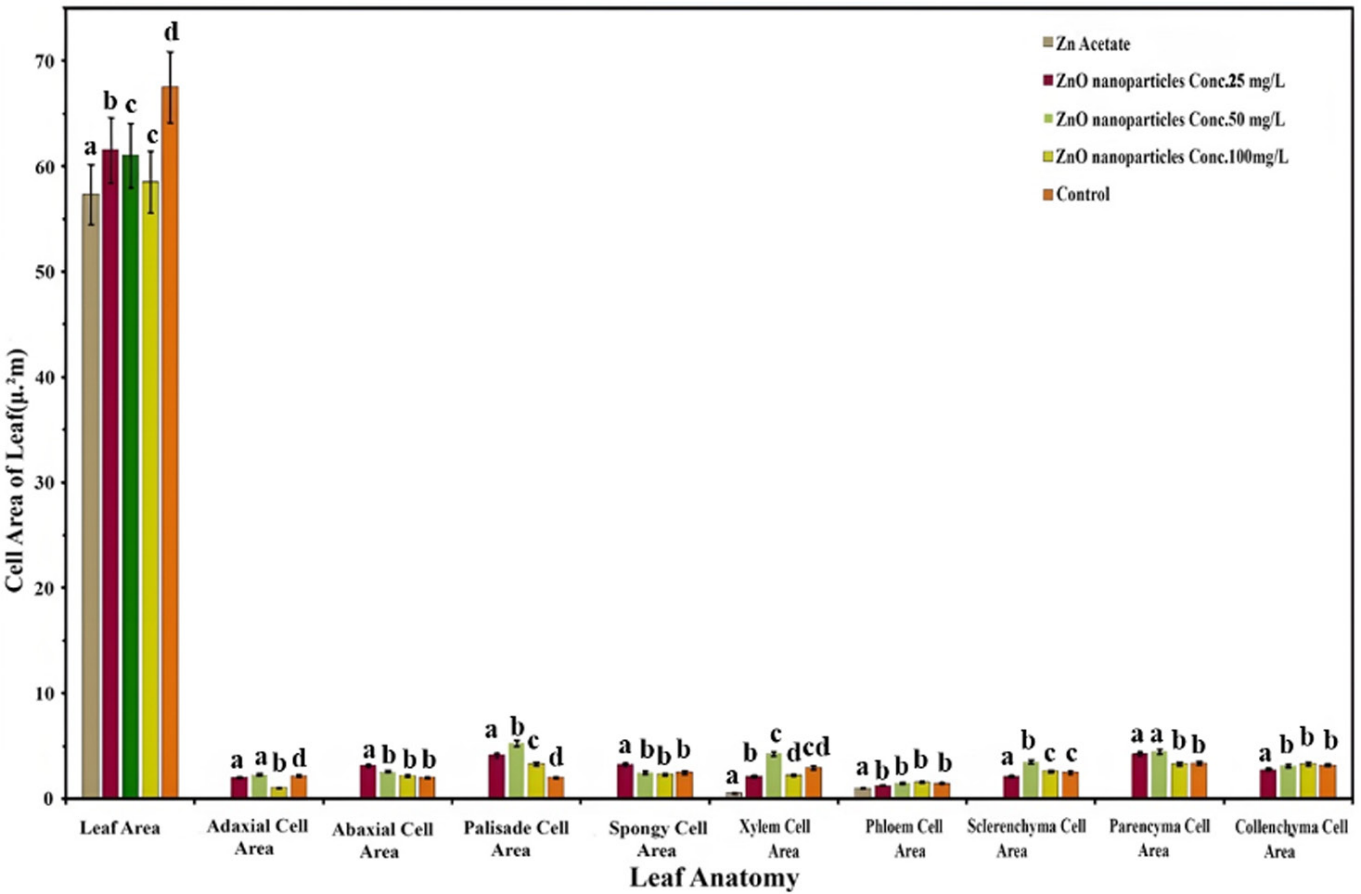
Effect of ZnONPs on the cell area of *Lactuca sativa* L leaf cells. The mean values at each point with different letters are significantly different according to ANOVA (*n* = 5, *p <* .05).

Data on the cell area of leaf tissues is discussed in Figure 7. Unlike leaf thickness parameters, the cell area of different leaf tissues is promoted by the foliar spray of ZnO NPs, except for the adaxial cell area, which squeezes with the significant increase in ZnONPs. The ANOVA results reveal that the application of 25 ppm denoted a sufficient increase in abaxial cell area and spongy cell area. Similarly, 50 ppm significantly promoted the palisade, xylem, sclerenchyma, and parenchyma cell areas, while 100 ppm slightly encouraged the phloem and collenchyma cell areas.

### 3.5 Antioxidative response of *L. sativa* to ZnO NPs

**FIGURE 8:**
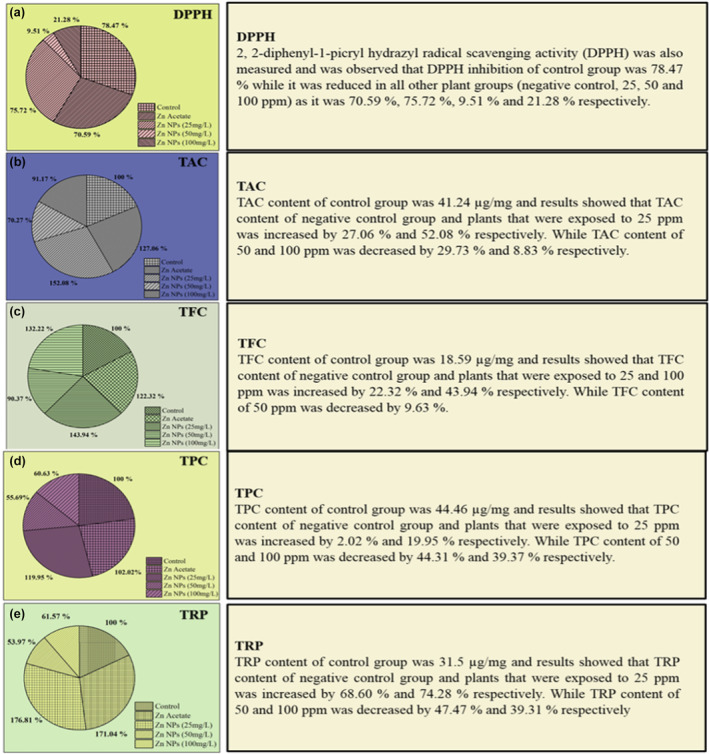
Antioxidative response of *Lactuca sativa* to ZnONPs: (a) 2,2‐Diphenyl‐1‐picryl hydrazyl radical scavenging activity (DPPH); (b) total antioxidant capacity (TAC); (c) total flavonoid content (TFC); (d) total phenolic content (TPC); (e) total reducing power (TRP). The mean values at each point with different letters are significantly different according to ANOVA (*n* = 5, *p <* .05).

The antioxidative responses of *L. sativa* were measured for 2,2‐diphenyl‐1‐picryl hydrazyl radical scavenging activity (DPPH), total antioxidant capacity (TAC), total flavonoid content (TFC), total phenolic content (TPC), and total reducing power (TRP) (Figure 8). The highest significant scavenging activity of DPPH was observed in the control group (78%) and the lowest activity was measured at a concentration of 50 ppm (9.5%) compared to all other concentrations, as shown in Figure 8a. Previous reports and current work provide evidence that zinc as a nanomaterial is very effective regarding the promotion of plant growth and boosting their immune system against disease resistance (Hasan et al., 2022; Hussain et al., 2022).

The TAC content of the control was 41.24 μg/mg, and the results showed that the TAC content of the negative control and plants that were exposed to 25 ppm increased by 27% and 52%, respectively, as shown in Figure 8b. While higher concentrations of ZnO NPs decreased leaf TAC content (Figure 8c). Similarly, the TFC content of the negative control (22%) and plants that were exposed to 25 ppm and 100 ppm was significantly increased by 44% and 32%, respectively, as shown in Figure 8d. The TPC and TRP content of the negative control and plants that were exposed to 25 ppm increased significantly by 2–69% and 20–74%, respectively, while these contents sharply declined with a gradual increase in ZnO NPs concentration (Figure 8e). Overall, the antioxidative activity of *L. sativa* considerably decreased at 100 ppm concentrations of ZnO NPs.

Nanotechnology is extensively practiced in agriculture for the improvement of crops yields through their use as nanofertilizers (Kalra et al., 2020) or nanopesticides (Bratovcic et al., 2021). Here, our study measured the morphological, anatomical, and antioxidative responses of *L. sativa* to different concentrations (0, 25, 50, and 100 ppm) of ZnO NPs applied as a source of Zn fertilizer. The hydroponic application of ZnO NPs with different concentrations has significantly improved plant growth and anatomical traits, but changes in the level of ZnONP concentration have also changed each plant trait response accordingly. These results showed better activity of ZnO NPs on plant vegetative traits, as previously reported (Sun et al., 2020). Most low and medium concentrations of ZnO NPs have a stimulatory effect on studied plant traits, while a higher concentration produces an inhibitory effect on the same trait (Zhang et al., 2020). This inhibitory effect might be due to toxicity induced by using higher concentrations of NPs. The 25–50 ppm concentration incremented plant fresh and dry weight, while the 100 ppm and zinc acetate treatments declined the fresh and dry weight accumulation as compared to the control. We have compared current research outcomes with previous reports, and it was found that plant growth was significantly improved by the application of 25–50 ppm concentration. Similarly, it was also reported before that the application of ZnO NPs to *Brassica oleracea* resulted in an enhancement of 37% in seed germination, 56% in root length, 16% in shoot length, 41% in weight of seedlings, 11% in number of leaves, 17% in plant height, and 24% in leaf area. In the current study, the ANOVA results reveal and confirm that the application of ZnO NPs documented an inconsistent trend in plant height, root length, and shoot length of *L. sativa* at concentrations of 25, 50, and 100 ppm. Similar trends have been observed in hydroponic culture and application of ZnO NPs promoted shoot length and root length in *Vigna radiata* and *Cicer arietinum* (Pavani et al., 2014) and shoot length and biomass accumulation in *Cyamopsis tetragonoloba* (Burman et al., 2013). It can be described that plants respond negatively to NPs if treated above the limited range due to elemental toxicity. It is demonstrated that shoot length of *Zea mays* (Itroutwar et al., 2020) and *Phaseolus vulgaris* (Salama et al., 2019) increased with an increase in NP concentration up to a limit; however, after certain concentrations (60 ppm), shoot length was found to decline. Recent reports also demonstrated that the influence of NPs differs from one plant to another. Treatment of the *Linum usitatissimum* (Sadak & Bakry, 2020) plant with ZnO and nano‐ZnO improved the studied growth parameters, biochemical aspects, and consequent yield. In another parameter, hydroponic culture application of ZnO NPs improved leaf, stem, and root weight as well as total leaf area when compared to the untreated plants. It can be suggested that the use of ZnO NPs might be an efficient way of increasing *Triticum aestivum* (Haq et al., 2020) growth and yield. Results of current study and previous research demonstrated both showed significantly stimulatory and inhibitory effects of ZnO NPs (Chaudhary et al., 2018). In the case of ZnO NPs, foliar application in *L. sativa*, the plant response changes with the change in concentration of ZnO NPs. The low and medium concentrations significantly promoted plant growth and biomass accumulation as compared to the higher doses of NPs and control treatments. At the anatomical stage, there are very few studies that recorded the responses of plants against NPs. The current study has also investigated the internal structural changes of *L. sativa* leaf, stem, and root, which exhibited the most noticeable response to hydroponic application of ZnONPs. In current results, it has been observed that there is an improvement in the thickness of the hypodermis at 25 ppm, the cortex at 100 ppm, and the pericycle at 25 and 100 ppm as compared to the control and Zn acetate, which is the negative control. The xylem and phloem thickness improved significantly at all concentrations of ZnONPs. At stem tissue cell area level, hypodermal cell area and sclerenchyma and collenchyma cell area at 50 and 100 ppm, epidermal cell area, cortex cell area, phloem, and pith cell area at 25 ppm showed significantly positive responses and improvements in their size. Current results are in corroboration with the already reported work (Abou‐Shlell et al., 2017), where Fe NPs have boosted *Moringa oleifera* stem and leaf anatomical measurements, that is, stem diameter, phloem and xylem tissue thickness, and vessel diameter. Furthermore, a wide range of certain NPs (Zn, Fe, Cu, and Si) applications have shown a positive impact on the histological characteristics of *Moringa oleifera* roots, that is, diameter of root, vascular cylinder, and xylem arch (Lawal et al., 2021). This positive effect on root anatomical traits may be due to NPs influencing cambium activity. The increase in cambium activity has been attributed to the increase in endogenous hormone levels, that is, cytokinin and auxin. All anatomical parameters of roots showed a negative response against zinc acetate; however, application of ZnO NPs favored some of the root anatomical traits. At root tissue thickness level, it was observed that application of ZnO NPs extended root diameter, endodermis thickness, phloem thickness and number of vascular bundles. At the root tissue cell area level, application of ZnO NPs improved the cell area of the epidermis, cortex, endodermis, phloem, parenchyma, and collenchyma. The cell areas of xylem and sclerenchyma showed a negative response at all concentrations of ZnO NPs. At the leaf tissue thickness level, it was observed that ZnO NPs stretched the thickness of cuticle, abaxial, adaxial, and palisade cells. The thickness of the midrib, lamina, spongy, xylem, phloem, and number of vascular bundles showed a negative response at all concentrations of ZnO NPs. The anatomical changes were studied in the cell areas of *Zea mays* (Ullah et al., 2020) and *Brassica oleracea* (Osuntokun et al., 2019) against the hydroponic applied Ag NPs and ZnO NPs. Both the NPs reduced the size of the vacuole, which ultimately diminished the cell turgidity and size. The negative effect on plant anatomical traits is due to the reduction in cell size that might have been attributed to lower osmotic cell enlargement or due to the radical alterations in ionic relationships that create hyper‐osmotic or hyper‐ionic stress and produce toxicity and plant cell demise. The application of TiO_2_ NPs favored leaf growth in *Zea mays*. Higher Zn accumulation (3–10 folds) was recorded in plant tissues supplemented with ZnO NPs (Pérez‐Hernández et al., 2021). Variations were also observed in total antioxidant power, total phenolic content, total flavonoid content, and total reducing power. All of these were increased at 25 ppm NPs. While free radical scavenging response was decreased at NPs treatments, NPs of Fe, Zn, and Cu significantly improved the antioxidant activity and total phenolic content of *M. oleifera*. An increase in antioxidant activity and total phenolic content with the application of different NPs is considered as a direct response to a boost in the rate of photosynthesis and its efficiency (Das et al., 2013). It might also be due to an increase in photosynthetic leaf area and a higher concentration of photosynthetic pigment (Lawal et al., 2021). It is due to the stimulatory mechanism of Zn as a nutrient when applied in the form of ZnO NPs that enhances its fast availability and easy uptake through foliar spray (Abomuti et al., 2021; Hasan, Mustafa, et al., 2018). In previous reports, biologically synthesized FeONPs enhanced plant antioxidative activity, and are an efficient source of Fe (Jafari & Hatami, 2022; Mohammadi et al., 2018). Application of AgNPs increased total phenolic content by 18%, flavonoid content by 12%, free radical scavenging activity by 7%, total phenolic content by 12%, and total reducing power by 10%. Seedlings taking Ag + ions showed an increment of 18% in total phenolic content and 12% in total flavonoid content, whereas under Ag NPs, 7% free radical scavenging activity, 12% total phenolic contents (TPC), and 10% total reducing power were increased. Overall, results showed that the ZnO NPs positively influence various morphological traits, including plant height, leaf area, and biomass accumulation, with concentrations of 25–50 ppm exhibiting the most significant improvements. However, the higher concentrations of ZnO NPs and zinc acetate treatments lead to decreased growth parameters. The concept of the present research revolved around the underlying mechanisms driving these morpho‐anatomical changes, such as Zn ion‐mediated physiological processes and nanoparticle‐mediated stress responses.

## 4 CONCLUSIONS

Zinc is an essential plant nutrient and performs multiple functions, including activating different enzymes. Plants face zinc deficiency due to their higher competition and lower availability in the soil. One of the sophisticated methods for its quick availability and easy access to plants is the foliar application of this nutrient. As the plant leaves have numerous microscopic openings that allow selective entry into the leaves, it is extensively imperative to synthesize NPs and optimize their dose for targeted application in plants. Application of ZnO NPs significantly improved *L. sativa* growth, anatomical, and antioxidative performance at 25–50 ppm. However, the 100 ppm zinc acetate proved fatal and inhibited plant activities. Future studies are needed to investigate the *L. sativa* response to foliar‐applied ZnO NPs in open field trials under varying agro‐climatic conditions.

## AUTHOR CONTRIBUTIONS

**Murtaza Hasan:** Conceptualization (equal); supervision (equal); writing – original draft (equal). **Tuba Tariq:** Data curation (equal); investigation (equal). **Ghazala Mustafa:** Investigation (equal); methodology (equal); software (equal). **Emad A. A. Ismail:** Supervision (equal); writing – original draft (equal). **Fuad A. Awwad:** Funding acquisition (equal); supervision (equal). **Mehrnaz Hatami:** Software (equal); writing – review and editing (equal).

## CONFLICT OF INTEREST STATEMENT

All the authors declared that there will be no conflict of interest.

## ETHICS STATEMENT

All methods performed in this study were in compliance with the relevant institutional, national, and international guidelines and legislation.

## CONSENT FOR PUBLICATION

Not applicable.

## Supporting information


**Figure S1.** Hand sowing of *Lactuca sativa* seeds in seedling tray containing coconut peat.
**FIGURE S2.** Germination of *Lactuca sativa* seeds in seedling tray placed in natural sunlight.
**FIGURE S3.** Germinating seedlings of *Lactuca sativa* after 7–14 days of sowing.
**FIGURE S4.** *Lactuca sativa* plant (a) Control (b) Zinc acetate (c) 25 mg/L (d) 50 mg/L (e) 100 mg/L after 35 days and ready to harvest.
**FIGURE S5.** Harvested plants of *Lactuca sativa* after flooding of ZnO NPs for 35 days.
**FIGURE S6.** (a) Plant Height (b) Area of Leaves (c) Number of Leaves of *Lactuca sativa* L.
**FIGURE S7.** (a) Shoot Length (b) Root Length of *Lactuca sativa* L exposed to different concentrations of ZnO NPs.
**FIGURE S8.** (a) Water Content (b) Dry Weight (c) Fresh Weight of *Lactuca sativa* exposed to different concentrations of ZnONPs.
**FIGURE S9.** ANOVA tables showing significant results.

## Data Availability

All the data are presented in the form of figures and tables.
