# Supplementary material for: Biogenic zinc oxide nanoregulator determines the quantitative analysis of morpho‐anatomical and antioxidant capacity in Lactuca sativa L
Source: Food Sci Nutr. 2024 Aug 18;12(10):7954–67. doi: 10.1002/fsn3.4261 (PMC11521655; doi:10.1002/fsn3.4261)

**Supporting Information**

**Biogenic Zinc Oxide Nanoregulator determine the Quantitative Analysis of Morpho-Anatomical and Antioxidant Capacity in Lactuca sativa L*.***

**Murtaza Hasan^1^, Tuba Tariq^2^, Ghazala Mustafa^3,4^*, Emad A. A. Ismail^5^, Fuad A. Awwad^5^, Mehrnaz Hatami ^6,7^***

^1^Department of Biotechnology, Faculty of Chemical and Biological Sciences, The Islamia University of Bahawalpur, 63100, Pakistan

^2^Department of Biochemistry, Faculty of Chemical and Biological Sciences, The Islamia University of Bahawalpur, 63100, Pakistan

^3^Key Laboratory of Horticultural Plant Growth and Development, Ministry of Agriculture, Department of Horticulture, Zhejiang University, Hangzhou 310058, China

^4^Department of Plant Sciences, Faculty of Biological Sciences, Quaid-i-Azam University, Islamabad 45320, Pakistan

^5^Department of Quantitative Analysis, College of Business Administration, King Saud University, P.O Box 71115, Riyadh 11587 Saudi Arabia

^6^Department of Medicinal Plants, Faculty of Agriculture and Natural Resources, Arak University, Arak, 38156-8-8349, Iran.

^7^Institute of Nanoscience and Nanotechnology, Arak University, 38156-8-8349, Arak, Iran.

*Corresponding author: [m-hatami@araku.ac.ir](mailto:m-hatami@araku.ac.ir)

Dr Ghazala Mustafa. [mghazala@qau.edu.pk](mailto:mghazala@qau.edu.pk)

## Morphological study of *Lactuca sativa*

Seeds of *Lactuca sativa* plants were obtained from market at Lahore, Pakistan and were sown by hand in a seedling tray consisting of peat moss as an organic medium. Water was sprinkled on peat moss and seedling tray was kept on a reservoir containing water to ensure the moisture required for seed germination. Seedling tray was kept in sunny areas and temperature was between 18-26 ℃.


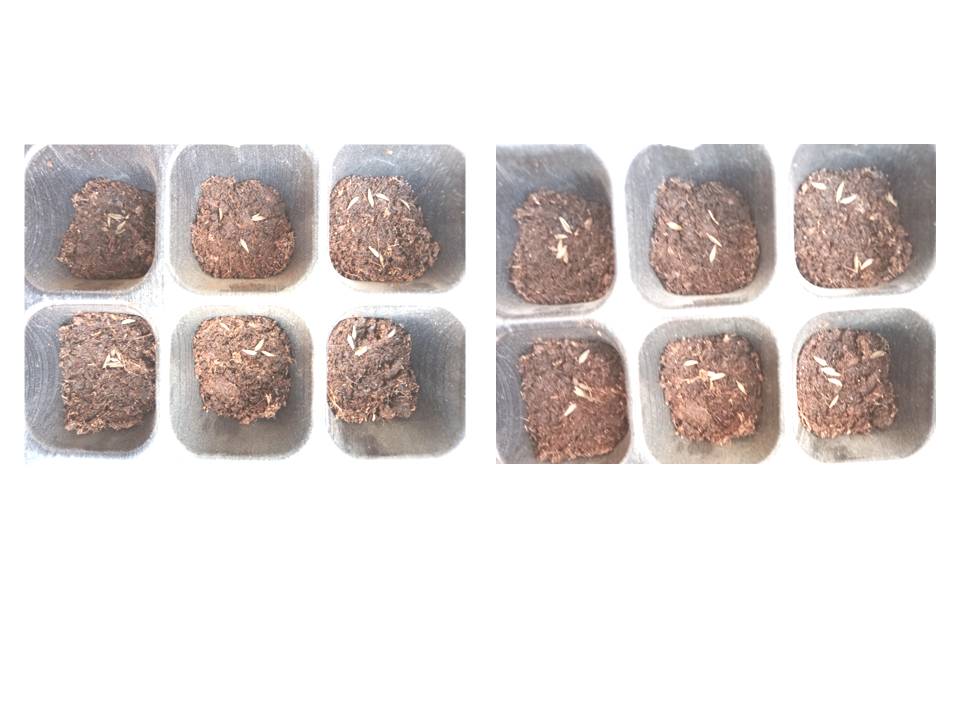


Figure S1. Hand sowing of *Lactuca sativa* seeds in seedling tray containing coconut peat.

The germination of seed started after a week and on organic medium, water was sprinkled everyday according to need for germination.


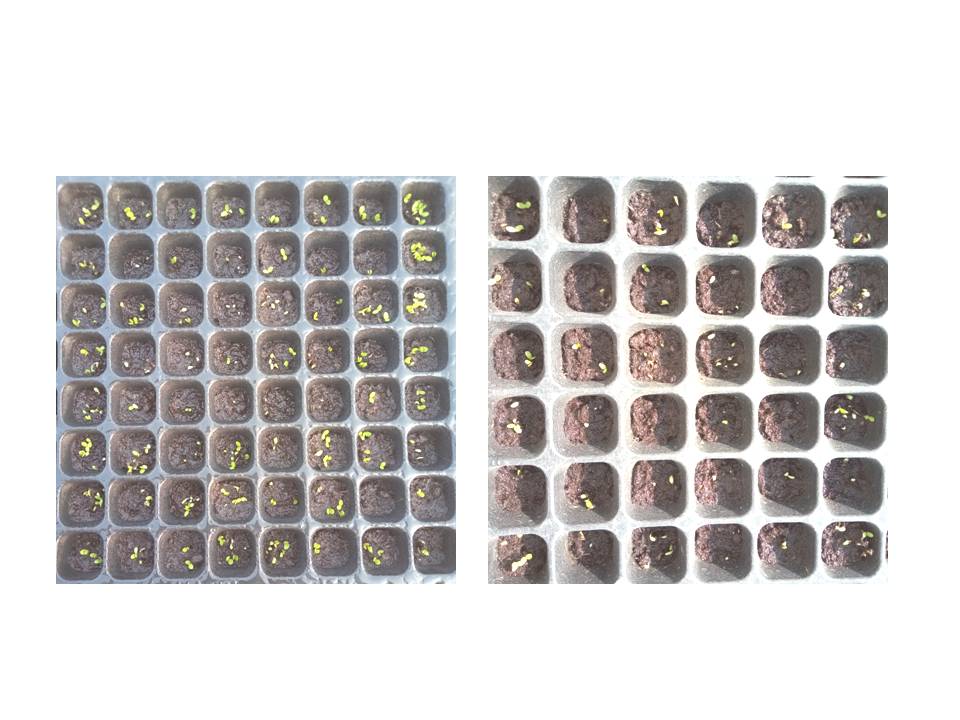


Figure S2. Germination of *Lactuca sativa* seeds in seedling tray placed in natural sunlight.

After about 5 cm height of seedlings, 10 seedlings were transferred to each pots carefully. These pots then were kept into a separate container that was filled with Hoagland’s solution.


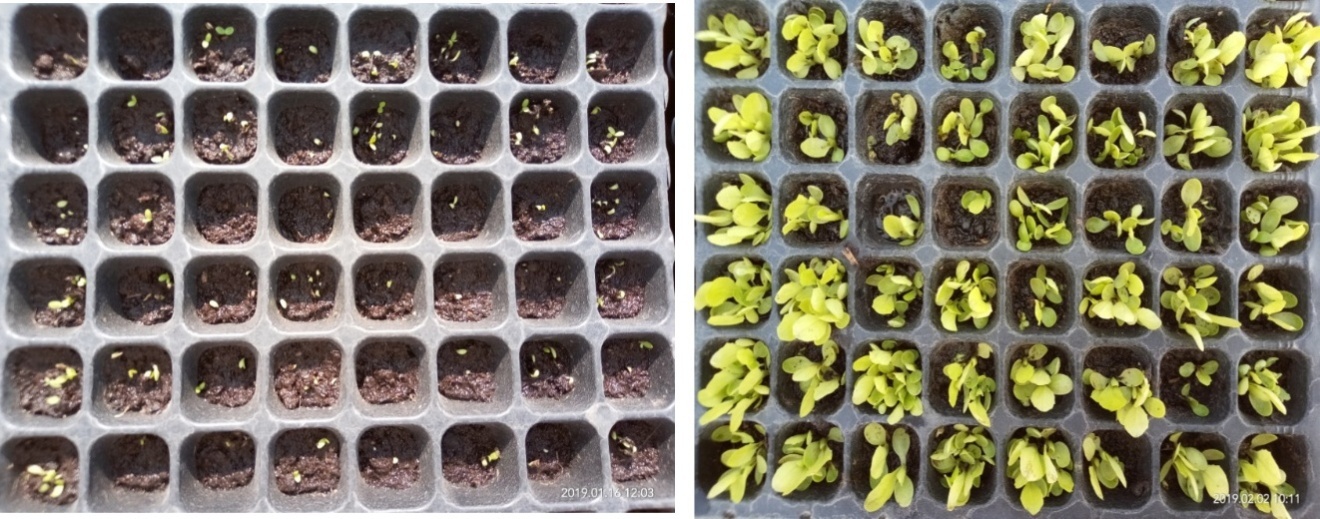


Figure S3. Germinating seedlings of *Lactuca sativa* after 7-14 days of sowing.

After 14 days seedlings were transferred to net pots and each pot then was placed in a container, there were 5 tests groups that were labeled. 15 ml of zinc oxide nanoparticles of different concentrations (25, 50 and 100 ppm) were supplied to every test group along with control group as well as negative control group was supplied with 0.01 M solution of zinc acetate. After 35 days treatment, *Lactuca sativa* plants were harvested.


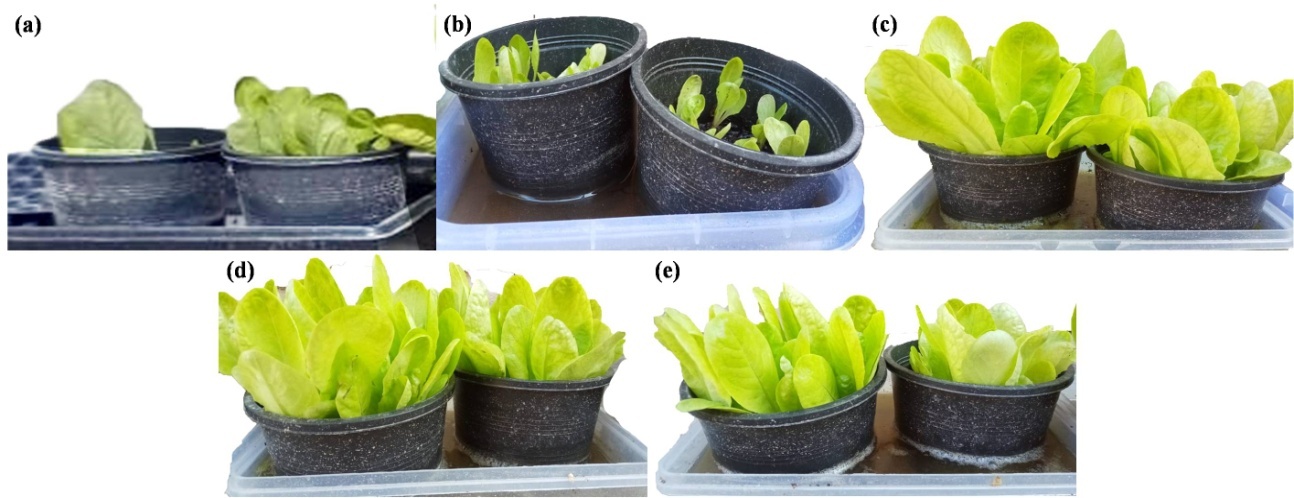


Figure S4. *Lactuca sativa*plant (a) Control(b) Zinc acetate (c) 25 mg/L (d) 50 mg/L (e) 100 mg/L after 35 days and ready to harvest.


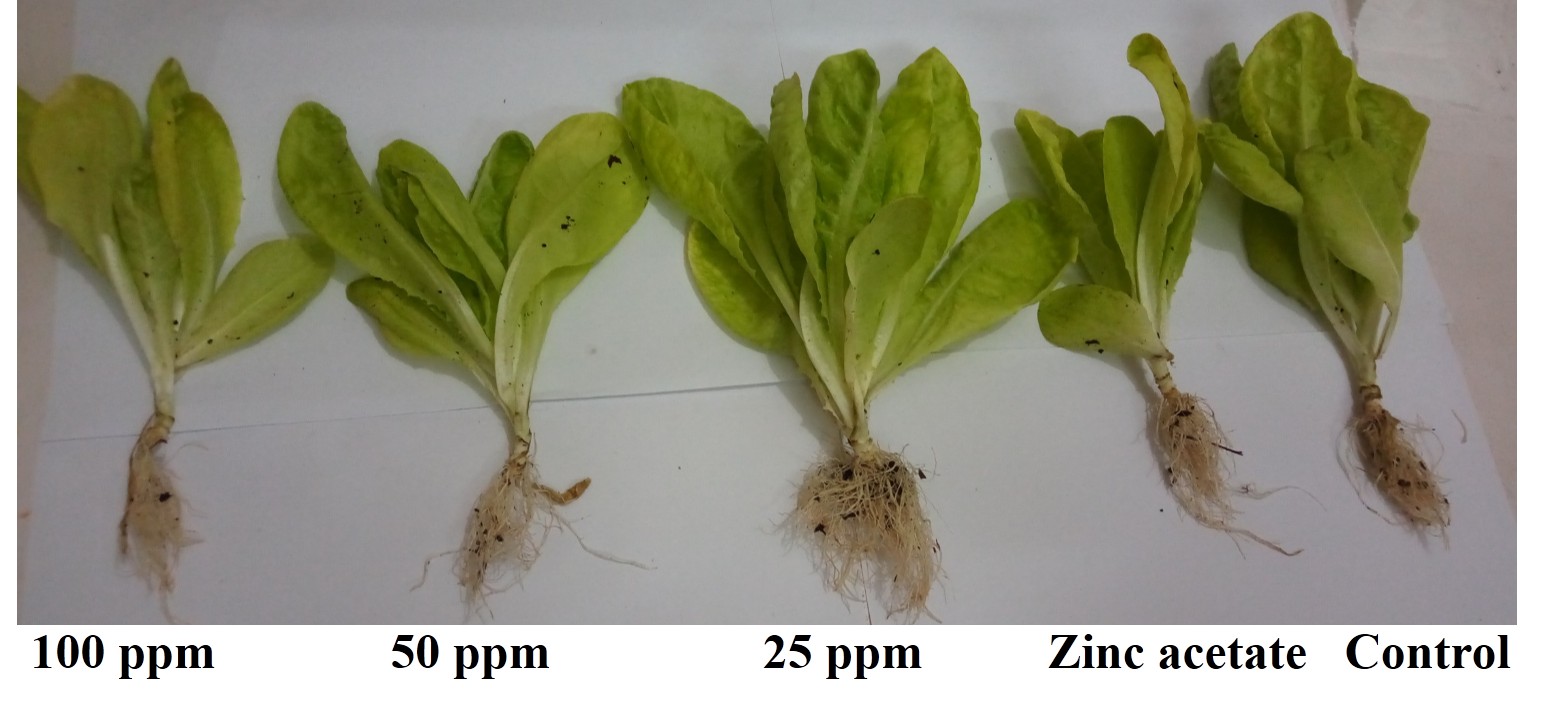


Figure S5. Harvested plants of *Lactuca sativa* after flooding of ZnO NPs for 35 days.


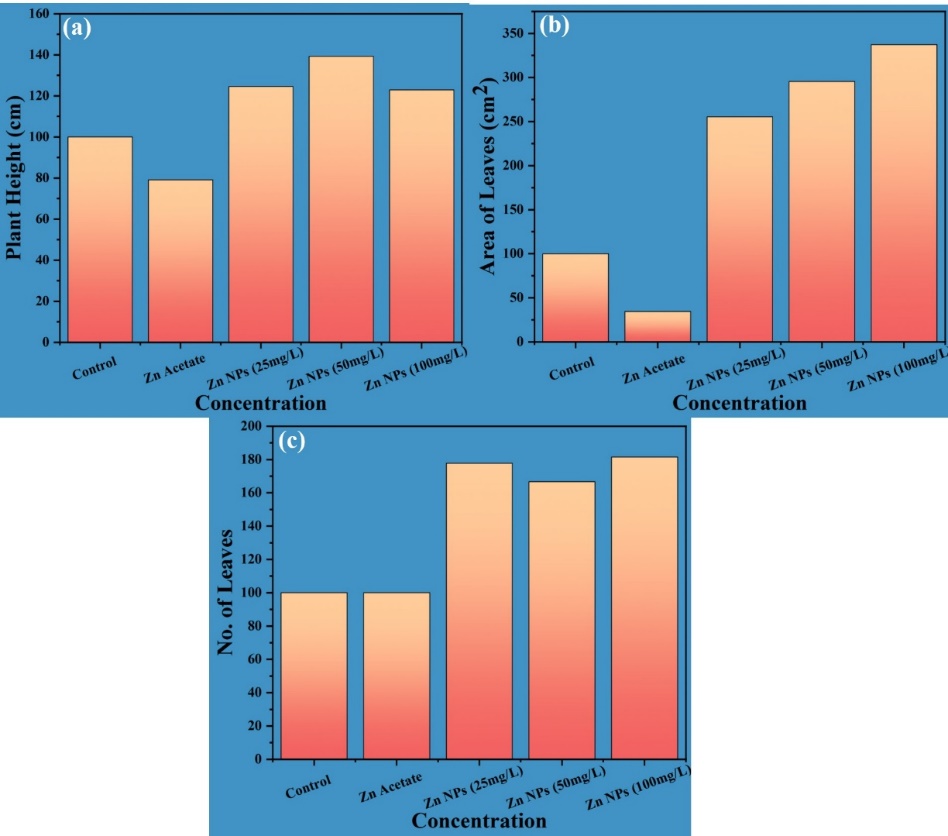


**Figure S6.** (a) Plant Height (b) Area of Leaves(c) Number of Leaves of *Lactuca sativa L.*


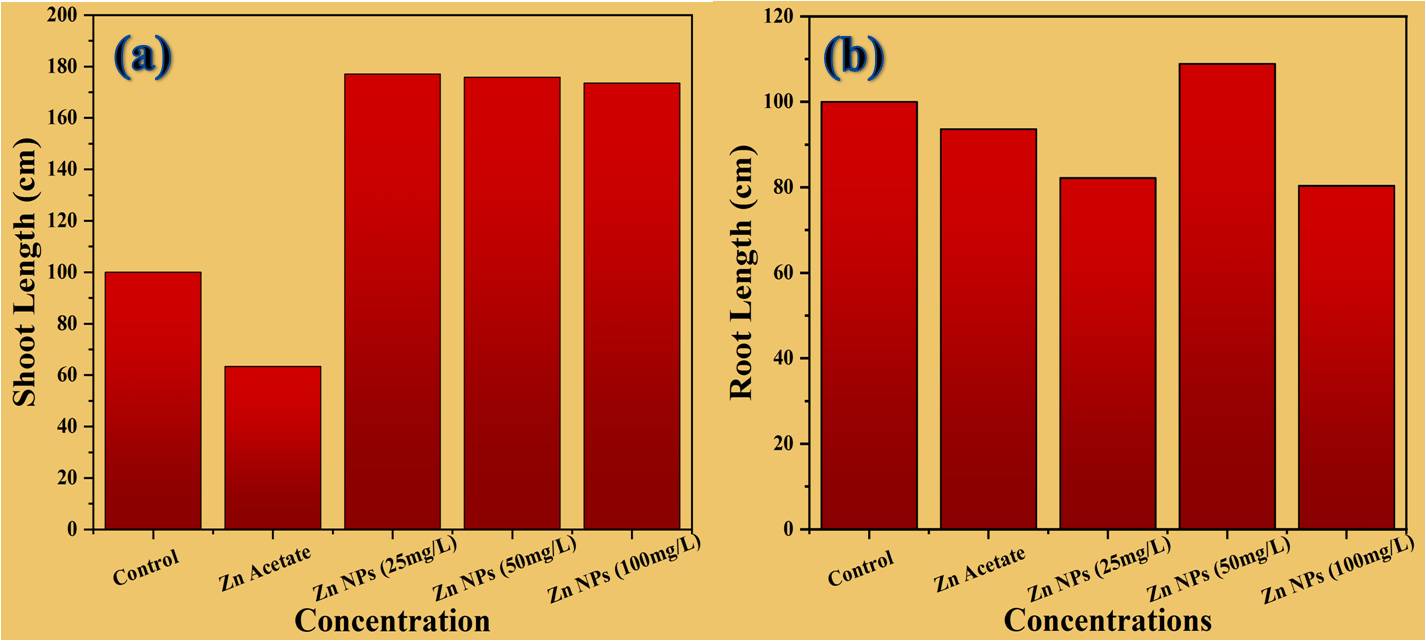


Figure S7. (a) Shoot Length(b) Root Length of *Lactuca sativa L* exposed to different concentrations of ZnO NPs.

**
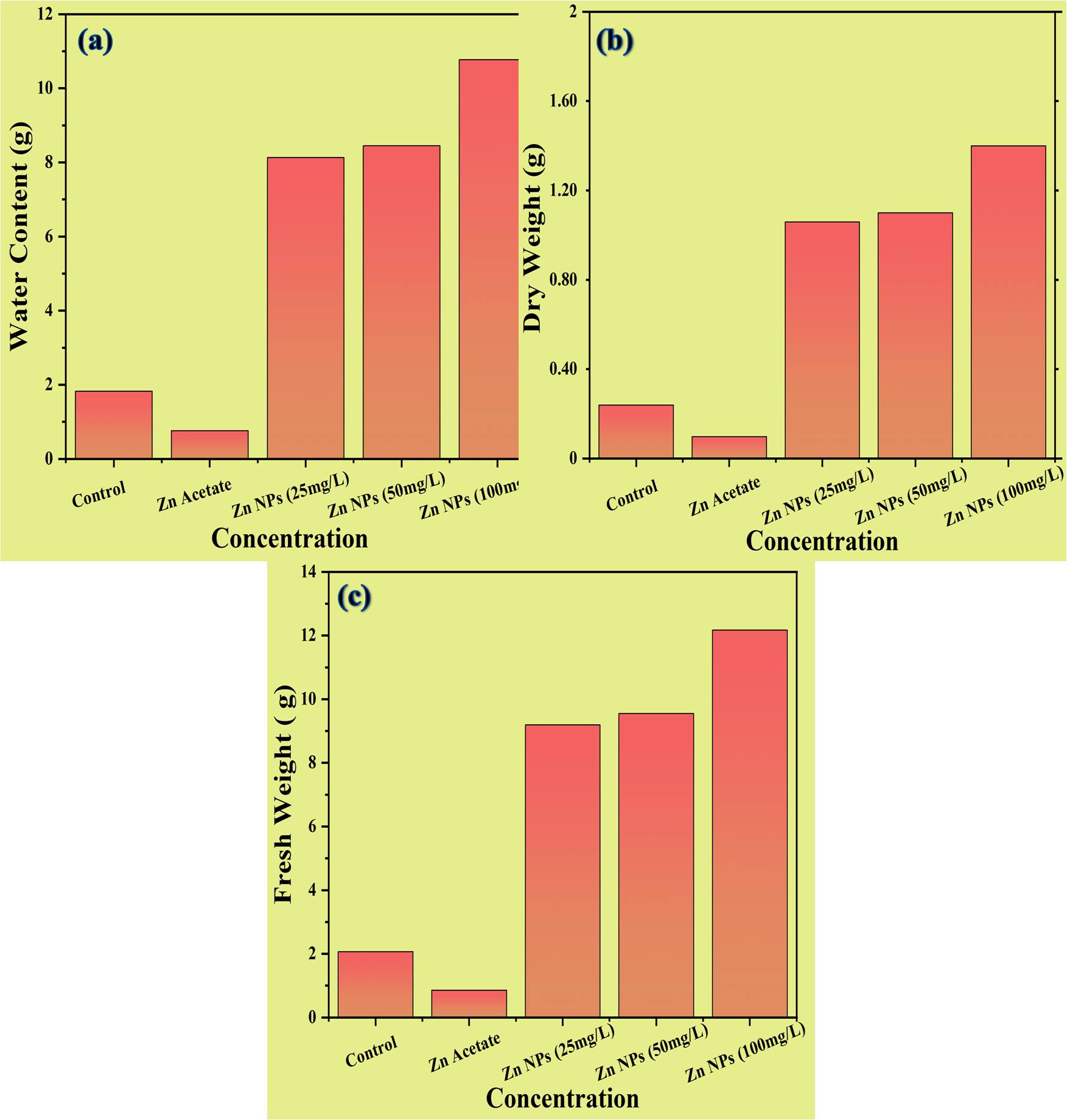
**

Figure S8. (a)Water Content(b) Dry Weight (c) Fresh Weight of *Lactuca sativa* exposed to different concentrations of ZnONPs.

Figure S9. ANOVA tables showing significant resuls.


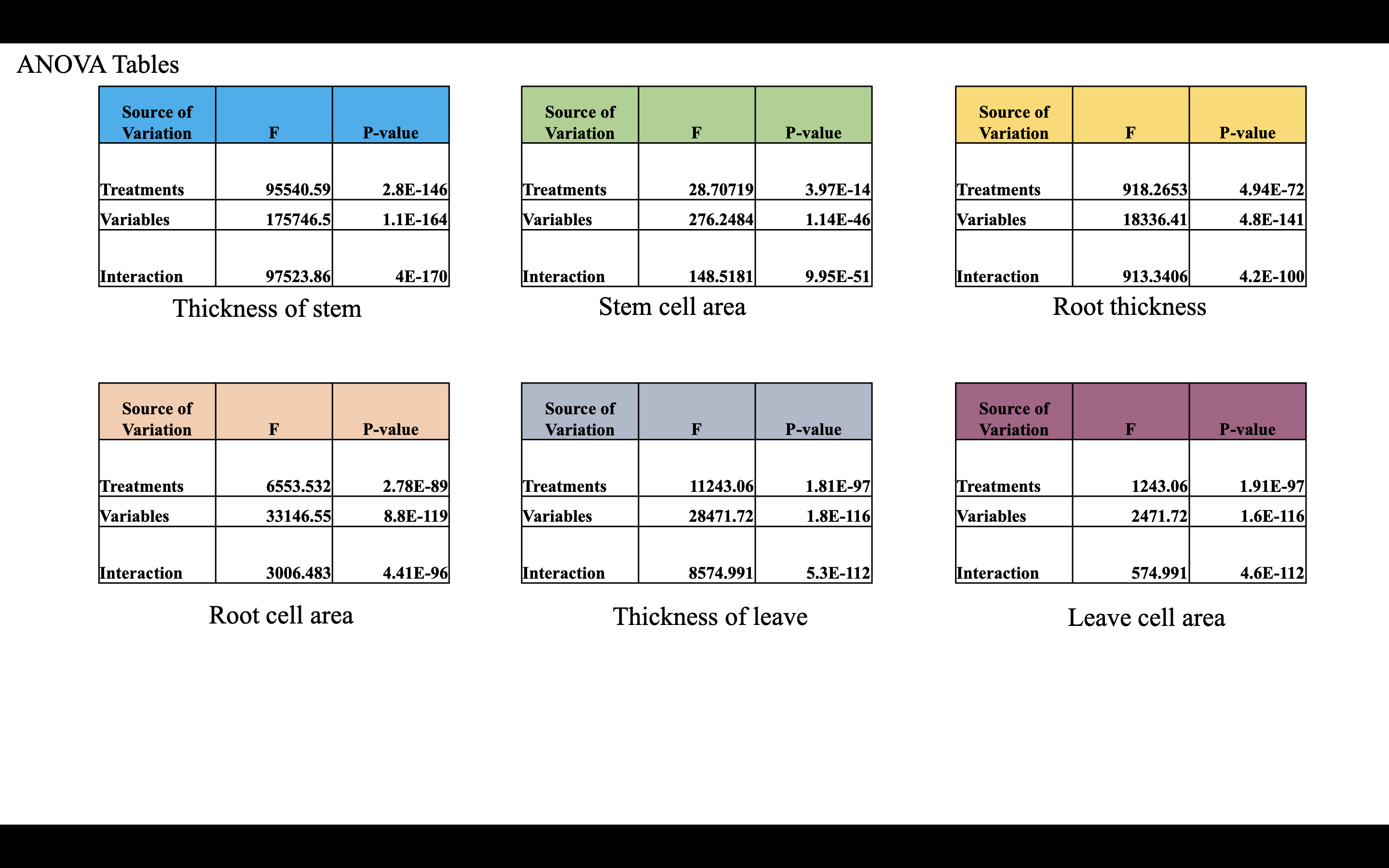

Supplement: Supplementary file 1 — Figure S1. Hand sowing of Lactuca sativa seeds in seedling tray containing coconut peat. FIGURE S2. Germination of Lactuca sativa seeds in seedling tray placed in natural sunlight. FIGURE S3. Germinating seedlings of Lactuca sativa after 7–14 days of sowing. FIGURE S4. Lactuca sativa plant (a) Control (b) Zinc acetate (c) 25 mg/L (d) 50 mg/L (e) 100 mg/L after 35 days and ready to harvest. FIGURE S5. Harvested plants of Lactuca sativa after flooding of ZnO NPs for 35 days. FIGURE S6. (a) Plant Height (b) Area of Leaves (c) Number of Leaves of Lactuca sativa L. FIGURE S7. (a) Shoot Length (b) Root Length of Lactuca sativa L exposed to different concentrations of ZnO NPs. FIGURE S8. (a) Water Content (b) Dry Weight (c) Fresh Weight of Lactuca sativa exposed to different concentrations of ZnONPs. FIGURE S9. ANOVA tables showing significant results. [file FSN3-12-7954-s001.docx]
